# Imidazolium Based Ionic Liquids: A Promising Green Solvent for Water Hyacinth Biomass Deconstruction

**DOI:** 10.3389/fchem.2018.00548

**Published:** 2018-11-21

**Authors:** Jitendra Kumar Singh, Rahul Kumar Sharma, Pushpal Ghosh, Ashwani Kumar, Mohammed Latif Khan

**Affiliations:** ^1^Metagenomics and Secretomics Research Laboratory, Department of Botany, Dr. Harisingh Gour Central University, Sagar, India; ^2^Department of Chemistry, School of Chemical Sciences and Technology, Dr. Harisingh Gour Central University, Sagar, India

**Keywords:** water hyacinth, lignocellulosic biomass, ionic liquids, pretreatment, hydrolysis, crystallinity index

## Abstract

Water hyacinth (WH) is a troublesome aquatic weed of natural and artificial water bodies of India and other tropical countries and causing severe ecological problems. The WH biomass is low in lignin content and contains high amount of cellulose and hemicellulose, making it suitable material for conversion into liquid fuels for energy production. This study highlighted that, how different imidazolium based ionic liquids (ILs) [1-alkyl-3-methylimidazolium bromide, [C_n_mim]Br (*n* = 2, 4, 6, 8, and 10)] with tunable properties can be employed for the degradation of WH biomass. Different characterizations techniques, such as XRD, FT-IR, SEM, and DSC are used to unravel the interplay between ILs and the biomass. In this study, it is observed that [Emim][Br] pretreated samples have maximum crystalline value (Crl = 26.38%) as compared to other ionic liquids pretreatments. FTIR data showed the removal of lignin from WH biomass by 12.77% for [Emim][Br] and 10.74% for [Edmim][Br]. SEM images have proven that [Emim][Br] pretreatment have altered the structure of biomass the most. Our results proved that IL pretreatment is a promising approach for effective treatment of WH biomass and causes high levels disruption of cellulose structure.

## Introduction

The limited supply of fossil based fuels in recent time has become a serious concern globally. Further, their associated negative impacts of fossil fuels on global climate are also becoming environmental concerns in many countries. Biofuels *in lieu of* fossil fuels has drawn a tremendous attention as a source of renewable, and clean energy (Kumar et al., 2010; Kumar and Sharma, 2011; Singh et al., 2014a; Raghunandan et al., 2018). Therefore, the search of alternative renewable energy source for biofuel production has expanded (Rezania et al., 2015). Lignocellulose is most abundant, renewable, cost effective, carbon neutral, non-edible plant material (Saini et al., 2015) and can be used for bioethanol production (Zabed et al., 2016; Singh et al., 2018; Vyas et al., 2018). Lignocellulosic materials for biofuels production are of much interest due to its low cost and high availability.

Till date, many plants biomass have been explored for the production of advanced biofuel by passing through three different steps; pretreatment, saccharification and fermentation (Sindhu et al., 2016; Kothari et al., 2017). Lignocellulosic biomass is composed of crystalline cellulose fibers which are embedded in a solid matrix of lignin and hemicelluloses that restrict the entry of microbial enzymes and also provide a barrier to microbial attack. Among all component of biomass, lignin is a highly branched, aromatic polymer, composed of phenylpropanoid units that serve as the glue that binds cellulose and hemicellulose, imparting rigidity and microbial resistance to lignocellulose (Chandra et al., 2007). Therefore, a proper pretreatment is required during the bioconversion process to disrupt the hydrogen bonds in crystalline cellulose to remove lignin and hemicellulose that surrounds the cellulose fibers and increase the porosity and surface area for enhanced enzymatic hydrolysis (Singh et al., 2014b).

**Graphical Abstract:**
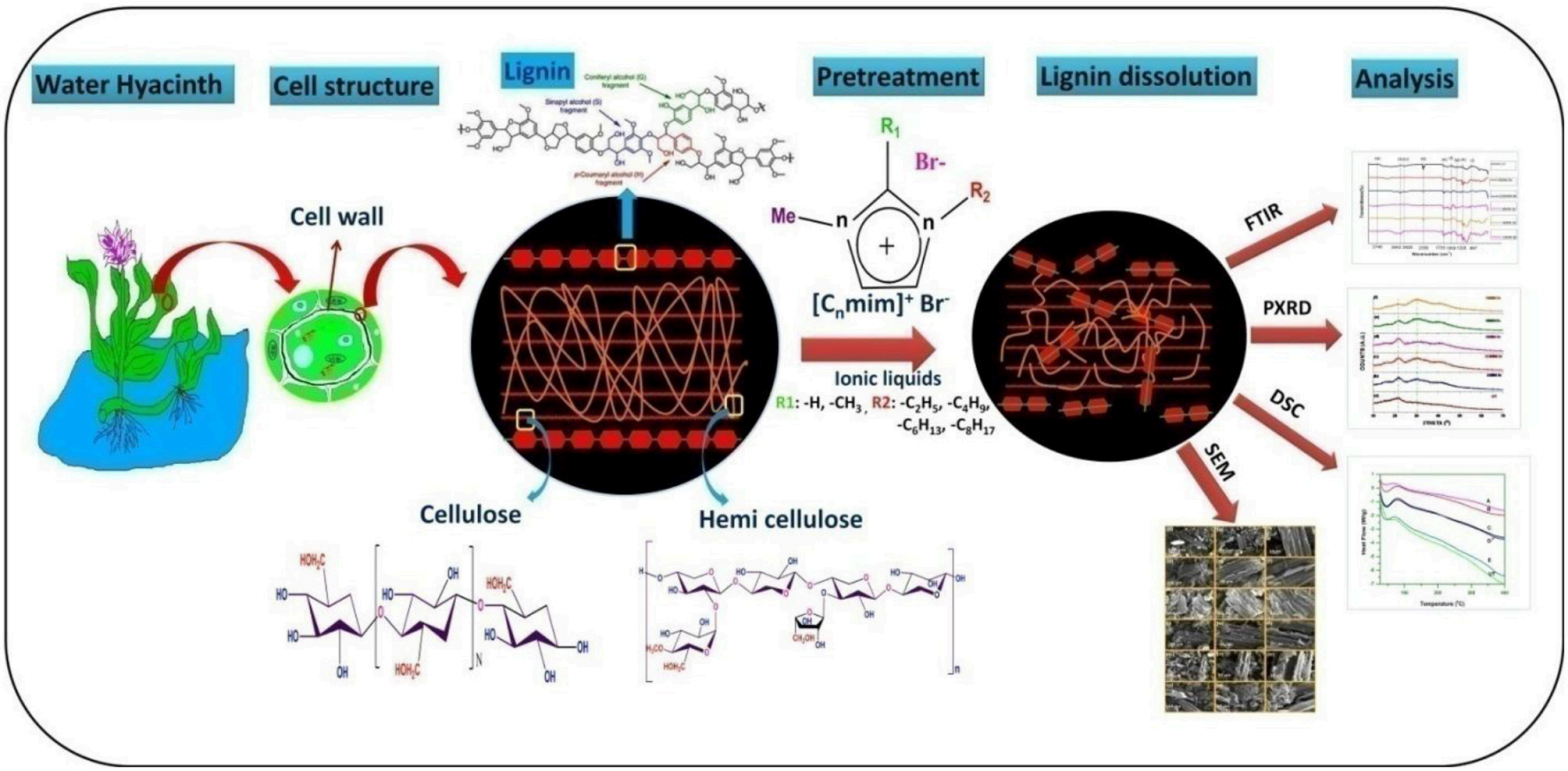


Different types of pretreatment methods have been used in the past to overcome the recalcitrance of lignocellulosic biomass which are dilute acid (Schell et al., 2003; Lloyd and Wyman, 2005), ammonia fiber expansion (Lau et al., 2008), hot water (Liu and Wyman, 2004), lime (Sierra et al., 2011) and organic solvent (Zhang et al., 2007). Among various pretreatment methods available for biomass hydrolysis, the ionic liquids (ILs) pretreatment has gained more popularity due to its ability to dissolve lignocellulose under simpler conditions (Menon and Rao, 2012). ILs are organic salts that usually melt below 100°C and often called as “green” and “designer” solvent (Sheldon, 2002). Application of ILs offers several advantages than volatile organic solvents, such as insignificant vapor pressure, good dissolving and extracting ability, wide liquid range, good thermal stability, excellent microwave-absorbing abilities, and they can be modified as per the requirement (Aid et al., 2016). In addition, physical and chemical properties of ILs can be effectively tuned by tuning the cation/anion combination; alkyl chain length, viscosity etc. and most importantly cellulose as well as the ILs can be recovered after the dissolution process with no toxic or odor emissions. The asymmetric nature of the cation part of ILs inhibits compact packing of the ions, and speedup the reactions that needs both high and low temperature. ILs display excellent characteristics in chemical processes with ability to dissolve polar and non-polar organic, inorganic, and polymeric compounds (Lee and Lee, 2005). Though, ILs is extensively used in catalysis, battery applications, inorganic synthesis, *f*-element separations etc., its application in biofuel production is still limited. Previously, several researchers have used variety of ILs for the dissolution of full lignocellulosic biomass using multistep processes followed by cellulose hydrolysis with acid or enzymes (Fort et al., 2007; Kilpeläinen et al., 2007; Li et al., 2009). Due to the protective feature of lignin, and its inability to undergo rapid degradation, different biomass pretreatment strategies have been focused on achieving a reduction in lignin contents. In dissolution process, ILs exhibit excellent physical characteristics including the ability to dissolve polar and non-polar organic, inorganic and polymeric compounds (Lee and Lee, 2005). They can dissolve the biomass due to the strong hydrogen bonding basicity of certain ions, such as Ac, Cl, Br, and NO_3_. These anions can extensively disrupt the hydrogen bonding interactions present in the three dimensional network of lignocellulose, leading to either dissolution of biomass or selected individual components based on the nature of the anion (Wei et al., 2012).

In this study, we have used ILs for the biomass hydrolysis of water hyacinth (*Eichhornia crassipes*; Family- Pontederiaceae, related to Liliaceae family) and analyzed the structural changes by FTIR, PXRD, DSC and SEM. Water hyacinth (WH), is a free floating troublesome aquatic weed plant that originates from Brazil and Ecuador. This plant multiply asexually (stolons) as well as sexually (seeds), at 25–35°C temperature and difficult to control due to its fast multiplication and long dormancy up to 20 years (Rezania et al., 2015, 2017). Single plant may produce 140 million individuals with fresh biomass weighing 28,000 tons each year under optimum conditions and aggravate eutrophication of water bodies, increases in CO_2_ emission, increased BOD (Biological Oxygen Demand) of water bodies, block river ways, obstruct navigation and causes irreversible damage to ecological system. Generally plant biomass growing on land is composed of about 30–50% cellulose, 20–40% hemicelluloses and 15–30% lignin. On the other hand, in WH biomass the amount of lignin (10%) is less and cellulose (20%) and hemicelluloses (33%) is high (Bolenz et al., 1990; Gressel, 2008), with high growth rate and no competition with land plants has led WH to be suitable material for biofuel industry (Feng et al., 2017). Previously, report showed that, acid and alkali based pretreatment were most commonly used methods for the pretreatment of WH biomass. Though, ILs based method was less utilized for the biomass hydrolysis. Here in this study, we have used imidazolium based ILs with pendant alky chain length for WH biomass hydrolysis. The whole process of biomass hydrolysis is presented in graphical abstract. A special care has been taken to understand how the structures and intricate properties of ILs influence the biomass degradation.

## Materials and methods

### Procurement of biomass samples

The water hyacinth (WH) biomass collected from Lakhabanjara lake at Sagar (23.8388° N, 78.7378°E), Madhya Pradesh, India. Collected WH biomass was processed by following the method of Ganguly et al. (2013). The harvested WH biomass was washed with distilled water vigorously to remove all undesirable matters, such as sand particles and cut to get average particle size of 1–2 cm. The cut WH biomass was then dried in an oven with the temperature of 105°C for 24 h. Dried plant material was powdered by grinder mixture (Jaipan 1290–750 W) and sieved to 0.02 mm mesh size. Subsequently, WH biomass samples were stored in a air tight glass container in a freezer at −4°C to maintain the aseptic condition until subsequent experiments. The pulverized WH biomass was stored in sterile condition because micro-organism can modify the complex chemical structure or main organic groups that constitute the WH biomass.

### Synthesis of ionic liquids

**Figure 1:**
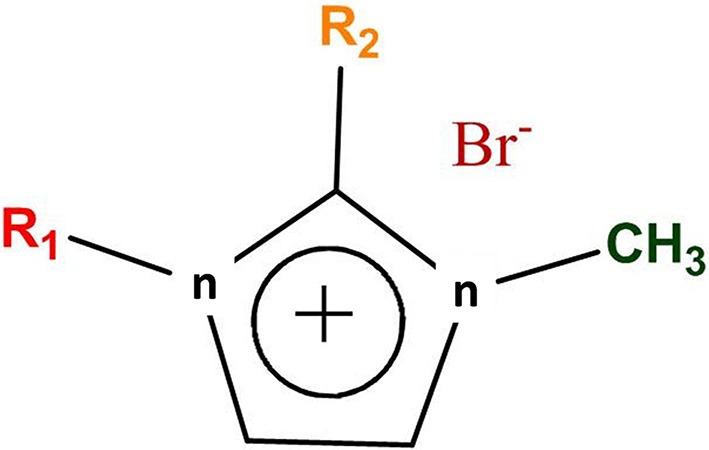
A general scheme of imidazolium based ionic liquids.

Five different ILs were synthesized and applied for WH biomass hydrolysis, which were based on imidazolium cation and bromide counter anion (Figure 1). The side chain of imidazolium cation is changed via varying the alkyl chain length. Furthermore, C (2) position of imidazolium ring is also substituted by methyl group in [Edmim]^+^[Br]^−^ in order to observe the role of acidic hydrogen on the degradation of cellulose of WH. The ionic liquids used in this study for the pretreatment of the WH sample are presented in Table 1.

**Table 1:** Types of as-prepared ionic liquids (ILs).

**S. No**.	**Ionic liquids (ILs)**	**Alkyl group (R_1_)**	**C(2) position (R_2_)**	**Abbreviations**
1	1-Ethyl-3-methyl imidazolium Bromide (IL1)	–C_2_H_5_	–H	[Emim][Br]
2	1-Ethyl-2,3-dimethyl imidazolium Bromide (IL2)	–C_2_H_5_	–CH_3_	[Edmim][Br]
3	1-Butyl-3-methyl imidazolium Bromide (IL3)	–C_4_H_9_	–H	[Bmim][Br]
4	1-Hexyl-3-methyl imidazolium Bromide (IL4)	–C_6_H_13_	–H	[Hmim][Br]
5	1-Octyl-3-methyl imidazolium Bromide (IL5)	–C_8_H_17_	–H	[Omim][Br]

### Chemicals

Tetra-methylammonium bromide (Loba Chemie, 98%), 1-methylimidazole (C_4_H_6_N_2_) (Alfa Aesar, 99.0%), 1,2-dimethylimidazole (Sigma Aldrich, 98%), Bromoethane (C_2_H_5_Br) (99.0%), n-Butyl Bromide (C_4_H_9_Br) (96.0%), n-Hexyl Bromide (C_6_H_13_Br) (99.0%), Octyl Bromide (C_8_H_17_Br) (98.5%) of Himedia brand were used. Ethyl Acetate (99.5%), Acetonitrile (99.5%), Dichloromethane (99.5%) were purchased from Loba Chemie and Silver Nitrate from Fisher Scientific brands were used. 1-ethyl-3-methylimidazolium bromide [Emim][Br], 1-ethyl-2,3-dimethylimidazolium bromide [Edmim][Br], 1-butyl-3-methylimidazolium bromide [Bmim][Br], 1-hexyl-3-methylimidazolium bromide [Hmim][Br] and 1-octyl-3-methylimidazolium bromide [Omim][Br] were prepared by modifying the previously reported methods (Ghosh and Mudring, 2016; Kumar et al., 2017). Synthesis procedure of the above mentioned ionic liquids (ILs) in detail is followed as:

#### 1-Ethyl-3-Methyl imidazolium Bromide [Emim][Br]

Modified procedure, 12.376 ml of bromo ethane (0.166 mol) and 10 ml of N-methyl imidazole (0.126 mol) were taken in a 250 ml round bottom flask and refluxed at 40°C for 4 h under Ar atmosphere in order to get complete reaction. Obtained product was allowed to attain the room temperature (RT) and product was washed twice with ethyl acetate. Resultant product was vacuum dried at room temperature for 24 h to obtain white solid product and checked by ^1^HNMR and ^13^C NMR.

#### 1-Ethyl-2,3-Methyl Imidazolium Bromide [Edmim][Br]

By using the similar procedure as stated above, [*Edmim*][Br] was synthesized, except 0.126 mol of 1, 2-dimethyl imidazole (Sigma Aldrich, 98%) was used instead of N-methyl imidazole.

#### 1-Butyl-3-Methylimidazolium Bromide [Bmim][Br]

This IL was synthesized through the previously reported method in which (0.166 mol) 1-bromo butane was added drop wise to (0.126 mol) 1-methyl imidazole in ice bath for 30 min with stirring. After that, flask was covered with aluminum foil and kept the reaction for 96 h at room temperature. Obtained product was re-crystallized from acetonitrile and vacuum dried at room temperature. The as-obtained product is checked with ^1^H NMR and ^13^C NMR.

#### 1-Hexyl-3-Methylimidazolium Bromide [Hmim][Br]

Modifying the previous literature procedure, 10 ml of N-methyl imidazole (0.126 mol) and 50 ml acetonitrile was taken in 250 ml round bottom flask, followed by drop wise addition of 23.22 ml (0.166 mol) of n-bromo-hexane in ice bath. After that reaction was allowed to be taken place at 60°C for 12 h. Obtained product was cooled at room temperature and washed with ethyl acetate two times. Obtained product was vacuum dried till golden yellow liquid being appeared.

#### 1-Octyl-3-Methylimidazolium Bromide [Omim][Br]

By following similar procedure, 1-octyl-3-methyl imidazolium bromide was synthesized. The required concentration of n-bromo-octane was taken according to its molecular weight and reaction was kept for 48 h. Golden yellow viscous liquid was obtained.

### Pretreatment of WH biomass

A 6% (w/w) WH biomass samples were pretreated with different ionic liquids as mentioned in Table 1. A WH biomass solution comprised of 0.3 g of biomass with 4.7 g of ILs in a 50 ml glass tube and this mixture was placed in water bath without stirring at 100°C for 1 h. After 1 h incubation, 35 ml of distilled water (DW) was added into the biomass/ionic liquids slurry and then centrifuged at 10,000 rpm for 10 min for the removal of IL. After that ILs pretreated WH biomass was washed at least four times with distilled water and solids were oven dried at 60°C till constant weight was obtained and then kept in a sealed plastic container at −4°C for further investigation.

### FTIR analysis

FTIR analysis was performed to observe the changes in structural and functional group in pretreated WH biomass. FTIR spectroscopy was carried out using a Fourier Transform Infrared Spectrophotometer (8400S SHIMADZU). The solid WH biomass samples (10 mg) were first mixed with the spectroscopic grade KBr (200 mg) (Merk, Germany) and ground to a fine powder in air by using a motor and pestle and then pressed into pellets for IR transmission studies. In each run, a background (pure KBr) was recorded (Chen et al., 2016). Biomass samples were scanned using an average 25 scan in the range of 400–4,000 cm^−1^ at spectral resolution of 2 cm^−1^. By using the FTIR data different values, such as lateral order index (LOI), hydrogen bond intensity (HBI) and total crystallinity index (TCI) (Hurtubise and Krassig, 1960; Nelson and O Connor, 1964; Nada et al., 2000) were calculated.

### Powder X-ray diffraction (PXRD)

PXRD was performed to observe the crystalline nature of the untreated and ILs pretreated WH biomass using a D8 Bruker Advance Cu-ray tube diffractometer (Cruz et al., 2013). The following parameter were set; Cu_Kα_ (λ = 1.54 A°) voltage (30 kV), and current (30 mA), 2θ scan range (0–60°), with scanning rate (2°/min) and a step size of 0.05° at room temp and sample were positioned on a quartz sample holder. The crystallinity value was obtained from the ratio between the intensity of the (002) peak (I_002_, 2θ = 22.5) and the minimum dip (I_am_, 2θ = 18.5) between the (002) and the (101) peaks as by using Equation (1) (Segal et al., 1959; Rodrigues et al., 2007).

(1) $$\begin{array}{l} \text{\%CrI }=\;\left[\left({\text{I}}_{002}-{\text{I}}_{\text{am}}/{\text{I}}_{002}\right)\right] \end{array}$$

Where I_002_ is the intensity of plane belongs to (002) and I_am_ is related to the amorphous structure.

### Scanning electron microscope (SEM)

The morphological changes in the pretreated biomass samples of WH before and after the ionic liquids pretreatment were observed by SEM. For sample preparation, the treated WH samples were fixed on stubs and gold layer coated using a Denton sputter coater system (Qiu et al., 2012). After gold coating samples were preserved in the desiccators till the analysis was performed. These samples were then imaged by FEI Nova Nano SEM™ 450 operated with an acceleration voltage of 15 kV and working distance of 5 mm. Different SEM images at various magnifications (500×, 1,000×, 3,000×) were recorded.

### DSC analysis

A differential scanning calorimeter having Simultaneous Thermal Analyzer (NETZSSCH): STA 449 F_1_ Jupiter was used with a N_2_ atmosphere in the range (25–450°C) at 10°C/min ramp. DSC curves were obtained with 3.3 mg untreated and pretreated WH biomass. The procedure used for DSC analysis was the same as described by Swatloski et al. (2002) and Bodirlau et al. (2010).

## Result and discussion

### FTIR analysis

**Figure 2:**
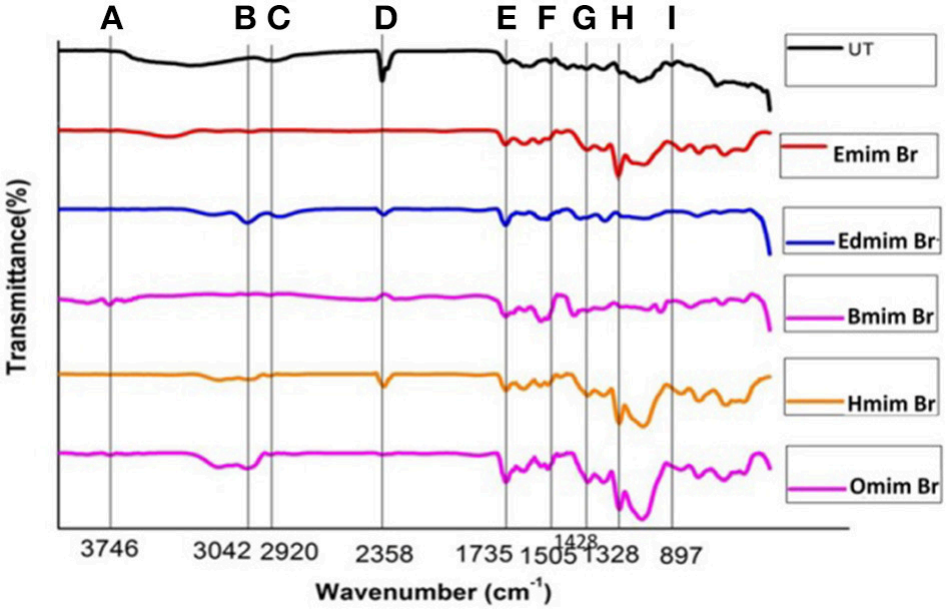
FTIR spectra of untreated and pretreated water hyacinth (WH) biomass: **(A)** 3746 cm^−1^; **(B)** 3042 cm^−1^; **(C)** 2920 cm^−1^; **(D)** 2358 cm^−1^; **(E)** 1735 cm^−1^; **(F)** 1505 cm^−1^; **(G)** 1428 cm^−1^; **(H)** 1328 cm^−1^; **(I)** 897 cm^−1^.

FTIR spectroscopy was used to affirm the changes in untreated and ILs pretreated WH biomass at structural level (Figure 2). Data related to the percentage relative changes in WH biomass and FTIR intensity value for different ILS pretreated samples are presented in Tables 2, 3, respectively. The main functional groups of the WH biomass components are cellulose, hemicelluloses, and lignin. The bands ranging in FTIR spectra from 3,000 to 3,500 cm^−1^, was assigned to the OH stretching vibration, which indicate the cellulose content in the sample. FTIR spectra at 3,348 cm^−1^ showed a significance reduction in the intensity of the band for ILs-pretreated WH sample, this peak is for O-H stretching vibrations and related to the hydrogen bonds in cellulose (Kumar et al., 2009) and changes related with this peak revealed changes in cellulose structure. Structurally, cellulose is having three hydroxyl groups which interact with other hydroxyl groups to form secondary valence bonds and responsible for cellulose crystallinity and chain structure by forming hydrogen bonding network. Changes in the peak intensity at 3,348 cm^−1^ showed the disruption of intermolecular and intra molecular hydrogen bonding (Alemdar and Sain, 2008; Yang et al., 2011). Comparative FTIR analysis revealed the efficacy of cellulosic hydrogen bond disruption by [Emim][Br] and [Edmim][Br] than the other ILs-pretreatment for WH biomass. Our results are supported by published report on pretreated switch grass (Li et al., 2010). Similarly the other band at 2,920 cm^−1^ is ascribed to C-H vibration for alkanes (Liu et al., 2006), and CH_2_–(C_6_)–bending vibration (Yang et al., 2011). The noticeable peak at 1,735 cm^−1^ which is ascribed to C = O stretching vibration in acetyl groups of the hemicelluloses was observed in untreated WH biomass (Karatzos et al., 2012), the intensity of this peak was very weak for the [Emim][Br] pretreated sample, as compared to the other ILs pretreated samples (Figure 2). The changes in the intensity of this peak could have been due to release of the acetyl groups in order of following treatments; [Emim][Br] pretreatment > [Edmim][Br] > [Bmim][Br] > [Hmim][Br] > [Omim][Br]. After IL pretreatment, this peak disappeared, indicated that some of hemicelluloses were removed in the dissolution process. The bands at 1,739 cm^−1^, and around 1,321–1,317 cm^−1^ are assigned to characteristic bending or stretching vibrations of the different groups from cellulose (Popescu et al., 2009).

**Table 2:** Percentage relative changes in WH biomass after ionic liquids pretreatments (Tiwari et al., 2018).

**Band position**	**Assignment**	**Pretreatment**					
		**UT**	**A**	**B**	**C**	**D**	**E**
3,332	O–H stretching (indicate the hydrogen bonds breaking in cellulose)		15.10	20.89	17.73	8.31	−8.71
2,906	C–H stretching (breaking of methyl/ methylene group of cellulose)		3.35	2.43	2.75	0.36	5.50
2,359	C–H methyl and methylene groups		8.70	5.63	4.14	−12.64	−15.46
1,730	Carbonyl bonds (it is linked with removal of lignin side chain)		2.26	1.62	1.34	−2.98	−5.85
1,510	C–C stretching (vibrations in the aromatic ring of lignin)		2.61	1.91	1.71	1.42	−5.46
1,420	CH_2_ scissoring at C(6) in cellulose		1.09	0.97	0.35	−4.91	−8.83
1,325	C–H deformation in hemicellulose		10.55	8.60	8.49	4.90	−9.41
1,058	C–O stretch		0.46	6.8	4.89	1.76	−15.80
897	C–O–C valence (vibration of β-glycosidic link)		4.82	3.51	1.55	−0.88	−7.12

Relative change (%) = 100* (intensity of UT solids – intensity of pretreated solids)/intensity of UT solids; where positive value of % relative change indicates reduction.

*UT, [Untreated]; A, [Emim][Br]; B, [Edmim][Br]; C, [Bmim][Br]; D, [Hmim][Br]; E, [Omim][Br]*.

**Table 3:** FTIR intensity value obtained for different ILs pretreated water hyacinth (WH) samples.

**Treatment**	**FTIR peaks (cm**^**−1**^**)**								
	**3,348**	**2,920**	**2,358**	**1,735**	**1,505**	**1,428**	**1,328**	**1,056**	**897**
Untreated	89.644	92.379	93.470	91.286	91.450	94.193	87.626	80.497	89.647
[Emim][Br]	99.578	99.488	99.250	93.311	95.628	91.101	91.465	85.464	99.604
[Edmim][Br]	99.529	98.402	98.267	96.677	97.718	92.090	98.017	97.910	97.197
[Bmim][Br]	99.345	99.281	99.576	94.541	93.946	93.177	95.854	96.183	96.094
[Hmim][Br]	99.358	98.996	99.317	94.421	96.162	93.580	92.734	83.454	95.288
[Omim][Br]	99.246	99.047	99.650	94.659	96.938	93.791	94.709	88.338	95.082

The bands between 1,595 and 1,230 cm^−1^ are assigned to characteristic bending or stretching of different groups of lignin. To observe the changes in lignin removal, following peaks at 1,328 and 1,505 cm^−1^ were observed, these peaks attributable to aromatic skeletal vibrations in lignin. Additionally, the peaks presented in the range of 1,428–1,458 cm^−1^ were allocated to the C–H deformation in lignin and carbohydrates (Pandey and Pitman, 2003). The peak noticed at 1,428 cm^−1^ is assigned to bending vibration of CH_2_ in cellulose and lignin. This band is strong in crystalline cellulose and weak in amorphous cellulose. Therefore, the amorphous cellulose in untreated, and ILs pretreated [Omim][Br], [Hmim][Br] and [Bmim][Br] samples is more than the samples treated by following ILs [Emim][Br] and [Edmim][Br]. The peaks located in the range 1,320–1,380 cm^−1^ in all untreated and pretreated ILs WH samples were ascribed to the bending vibration of C–H and C–O groups in the aromatic ring (Genestar and Palou, 2006). By applying different ILs pretreatment to WH biomass, we observed the decrease in intensity of the lignin linked peaks (peak at 1,514 cm^−1^ is associated with the aromatic skeletal modes of lignin and the peak at 1,328 cm^−1^ denotes the aromatic hydroxyl groups generated by the cleavage of ether bonds in lignin) which showed the effectiveness of the ILs pretreatment (Hsu et al., 2010). It was seen that the ILs pretreated WH biomass samples were delignified more in comparison to the untreated ones for the peak intensity changes at 1,328 and 1,514 cm^−1^ (Figure 2; Table 4). For example,[Emim][Br] pretreated WH biomass was delignified slightly more efficiently than the other ILs pretreatments. While, ILs pretreated samples showed the less intensity at 1,505 cm^−1^ over untreated one. The absorbance at 1,158 and 897 cm^−1^ correspond to C–O–C vibration in cellulose and hemicelluloses, and C–H deformation in cellulose, respectively (Pandey and Pitman, 2003). At 897 cm^−1^ the peak obtained showed changes with ILs used in the study. The band at 897 cm characterized by C–O–C stretching at β 1–4 glycosidic linkage, showed the amorphous nature of cellulose. The peak observed at 897 cm^−1^ was less intense in case of [Emim][Br] and [Edmim][Br] pretreated WH biomass compared to the other ILs ([Bmim][Br], [Hmim][Br], and[Omim][Br]) and untreated WH biomass samples. The presence of band at 897 cm^−1^ is strong and sharp that showed the presence of amorphous cellulose. So, from (Figure 2; Table 3) it is concluded that [Emim][Br] and [Edmim][Br] pretreated WH biomass samples have less amorphous cellulose than other ILs pretreated WH biomass samples used in this study.

**Table 4:** LOI, TCI and HBI index of untreated, ionic liquid treated water hyacinth (WH) samples.

**Samples**	**LOI**	**TCI**	**HBI**
	**1,420/891 cm^−1^**	**1,370/2,900 cm^−1^**	**3,338/1,334 cm^−1^**
Untreated	0.980	0.940	1.870
[Emim][Br]	1.570	0.997	1.291
[Edmim][Br]	1.450	0.980	1.383
[Bmim][Br]	1.389	0.971	1.537
[Hmim][Br]	1.237	0.965	1.649
[Omim][Br]	1.190	0.953	1.779

*LOI, lateral order index or crystallinity index; TCI, total crystallinity index; HBI, hydrogen bond intensity*.

We have compared the percentage changes in different spectra for untreated as well-pretreated WH biomass samples that are calculated by subtracting the intensity of the important peaks in the untreated WH biomass material from that of respective peaks in the pretreated WH biomass samples with different ILs (Table 2). The results of this calculation showed that the peak intensity at 1,510 cm^−1^ decreased by about 12.77% for the [Emim][Br] and by 10.74% for the [Edmim][Br]. Our results supported the efficacy of ILs pretreatment as appropriate method for removing lignin (Figure 2; Table 3).

This study showed the increased crystallinity index in the ILs pretreated WH biomass samples, which could have been due to the partial removal of hemicelluloses and lignin from the biomass.

The TCI, LOI and HBI for untreated and ILs pretreated biomass are shown in Table 4. For marking the changes in cellulose structure the crystalline and amorphous regions, were indicated by two peaks at 1,428 and 896 cm^−1^ denoted (O'Connor et al., 1958). During the ILs pretreatment of WH biomass samples these two absorption bands showed changes due to the vibrating nature of cellulose in crystalline region and amorphous area. Therefore, the ratio of the intensities of these two bands was defined as an empirical “crystallinity index,” and it was termed as the “Lateral Order Index” (LOI). Generally, decrease in LOI value indicate the decrease in crystallinity (Oh et al., 2005; Kljun et al., 2011). The ratio between the bands at 1,372 and 2,900 cm^−1^, also proposed by Nelson and O Connor (1964) to be the total crystallinity index (TCI), was used to evaluate the infrared crystallinity (IR) ratio.

The value LOI and TCI for the ionic liquid pretreated WH biomass samples are shown in Table 4. WH biomass pretreated with different ILs, showed high LOI and TCI value as compared to untreated sample, indicating that untreated sample possessed a lower crystallinity value. Therefore, WH biomass pretreated with different ILs are indicative of biomass with a higher crystallinity and more ordered structure of cellulose as represented by the higher values of given index LOI and TCI.

In this study, an empirical index HBI and LOI were used to interpret qualitative changes in crystallinity for all WH biomass samples, and HBI was used to study the changes of hydrogen bonding between certain hydroxyl groups in cellulose, generally crystallinity decreases with increasing HBI value (Oh et al., 2005). Siroky et al. (2010) reported that the two peaks at 3,338 and 1,336 cm^−1^ were closely associated with the crystalline cellulose and intra-, inter- molecular regularity. The TCI value is comparative to the crystallinity degree of cellulose in the biomass (Carrillo et al., 2004) and LOI is proportional to the degree of order in cellulose (Corgiè et al., 2011). Ionic liquids [Emim][Br] pretreated WH biomass presented the more TCI and LOI values, which is directly correlated to degree of crystallinity and more ordered structure of cellulose in comparison to other ionic liquids pretreated WH biomass. On the other hand, ionic liquids [Omim][Br] presented the lowest TCI and LOI values, which may indicate that the cellulose of this pretreated WH biomass is composed of more amorphous domains when compared with the other ionic liquids pretreated WH biomass. While ionic liquids [Edmim][Br], [Bmim][Br] and [Hmim][Br] pretreated WH biomass presented intermediate values. HBI value is another indicator of ordered nature of cellulose and high degree of intermolecular regularity. From Table 4, the decreasing HBI values for [Emim][Br] and [Edmim][Br] pretreated WH biomass samples recommended the increase in degree of intermolecular regularity during the conversion process.

### PXRD analysis

**Figure 3:**
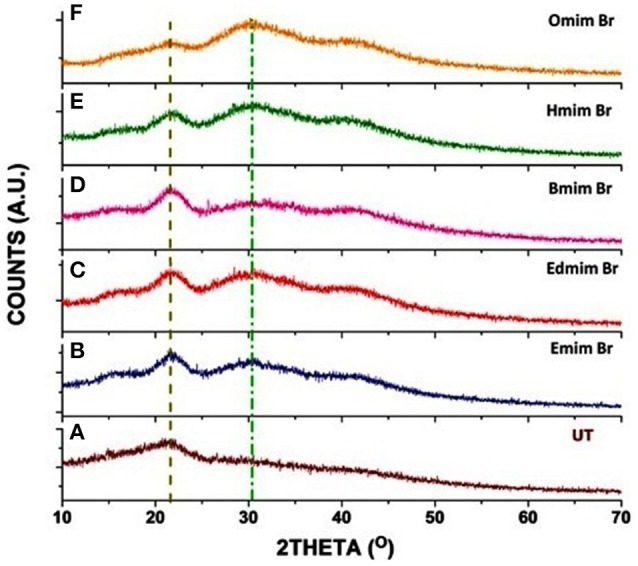
PXRD patterns of untreated and pretreated water hyacinth (WH) biomass samples: **(A)** (UT) [Untreated]; **(B)** [Emim][Br] treated; **(C)** [Edmim][Br] treated; **(D)** [Bmim][Br] treated; **(E)** [Hmim][Br] treated and **(F)** [Omim][Br] treated.

The Powder X-ray diffractometer (PXRD) was used to detect the effect of ILs pretreatment on WH biomass. The PXRD profile of untreated and ILs pretreated WH biomass have shown two well-defined peaks of cellulose around 2θ = 22.5° (for the 002 peak) and 2θ = 15° (for the 001 peak) (Figure 3). It is observed after the analysis that all the untreated and pretreated samples showed the typical PXRD patterns of cellulose. Our results of PXRD analysis of ILs pretreated WH biomass showed two peaks one with 2θ range at 21–23° corresponding to the crystallographic forms of cellulose and second broader peaks with 2θ range 15–19° for cellulose. In this study, ILs pretreatment induced some changes in the peak which was depicted in the PXRD patterns at the value of 2θ angle.

The untreated WH biomass revealed a lower crystallinity value (CrI = 18.63%) in comparison to the ILs pretreated samples. These ILs pretreated samples [Edmim][Br] (CrI = 25.41%) and [Emim][Br] (CrI = 26.38%). [Emim][Br] have maximum crystalline value as compared to other ILs pretreatments in this study (Table 5). Other study supported our findings, where they have mentioned that the increase in peak intensity could be due to the untreated raw fibers containing non-cellulosic amorphous constituents, such as hemicelluloses and lignin (Silvério et al., 2013; Mtibe et al., 2015). While in some cases the stem explosion pretreated biomass indicated increase in the value of this index (Tanahashi et al., 1983). The main reason behind increase in crystallinity is due to the removal of lignin and hemicelluloses fraction (amorphous substances) and not certainly due to changes in the crystalline structure of the biomass (Table 4).

**Table 5:** CrI value of water hyacinth (WH) biomass.

**S. No**.	**Treatments**	**Crystallinity index (%)**	**Increases (%)**
1	Untreated	18.63	–
2	[Emim][Br] treated	26.38	41
3	[Edmim][Br] treated	25.41	36
4	[Bmim][Br] treated	22.01	18
5	[Hmim][Br] treated	21.32	14
6	[Omim][Br] treated	20.73	11

These PXRD patterns generated here showed semi-crystalline substances with crystalline peaks and amorphous broad hump (Santos et al., 2013). In this study, it's clear that the CrI of ILs pretreated WH had a higher value than the untreated WH biomass. Zhao et al. (2011), in their study reported that crystalline structure of cellulose is more recalcitrant to microbial and enzymatic attack, compared to amorphous cellulose.

From PXRD patterns (Figures 3A–F) of the untreated and pretreated samples using various ILs, it can be evidently seen that how the ILs are playing a significant role in the dissolution of lignin of WH samples. Previously, it is illustrated that anion and imidazolium ring of IL plays a crucial role in the dissolution of lignin via hydrogen bonding and π-π stacking interaction, respectively (Hossain and Aldous, 2012). Here we have analyzed the influence of alkyl chain length on the interaction between imidazolium cation and lignin in which anion is same. The pendant alkyl chain (number of carbon atoms in alkyl group C = 2, 4, 6, and 8) length is increased to C-1 position of imidazolium cation. And the intensity of peak localized in the range of 15–25°, 2θ is substantially changing with alkyl chain length of IL. Here, intensity of peak centralized at 21.59° is gradually increasing up to [Bmim] Br (Figures 3B–D) however peak intensity is decreasing from [Hmim] Br and almost disappeared in the case of [Omim] Br (shown in Figures 3E,F). Further, hump like broad peak around 30–34° is simultaneously appearing as well-increasing with the alkyl chain length of imidazolium cations.

Moreover, it is clearly indicated that interaction between ILs and lignin molecules is dependent on the alkyl chain length of the cation. Longer the alkyl chain length more would be the steric hindrance. Consequently, lesser would be the attachment of IL cation over the surface of the WH; less amount of lignin will be dissolved in the IL. As the anion is common in case of all ILs, the role of anion on crystallinity value can be nullified.

Furthermore, the effect of IL is on the pretreatment sample can be quantitatively related to the crystallinity index (CrI) which is calculated using the Equation (1). The extent of dissolution of lignin can be indirectly determined through the CrI value. If the CrI value is decreasing with the alkyl chain length of the ILs, it means interaction between the IL and lignin will not be taken place to the significant extent. For instance, it is obtained highest for the sample pretreated with [Emim][Br] whereas this value is obtained very less for [Omim][Br] (20.73%) and least value is found for the untreated sample (18.63%). Same trend is also obtained for increasing the percentage of CrI of pretreated samples using different ILs with respect to the untreated sample (1) (as shown in Table 4), maximum value i.e., 41% is obtained for the [Emim][Br ] and minimum for the [Omim][Br] (20.73%). Crystallinity index value is observed greater for the [Emim] [Br]. Similarly FTIR results also support PXRD results. Because, LOI and TCI is greater for [Emim] [Br] than that of [Omim] [Br].

### SEM analysis

**Figure 4:**
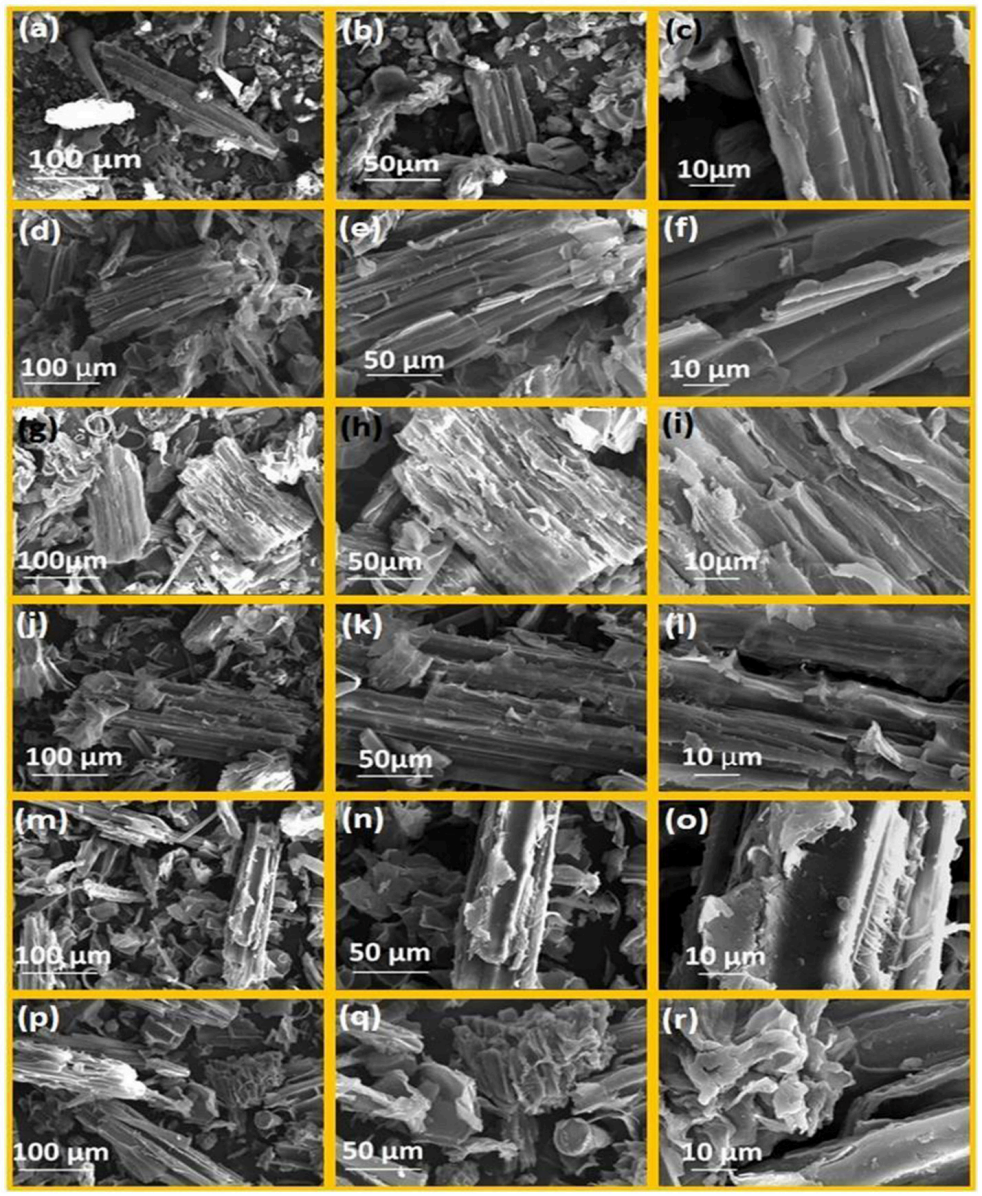
SEM images of water hyacinth biomass at following magnification (500×, 1000×, 3000×); **(a–c)** untreated biomass; **(d–f)** [Emim][Br]-pretreated water hyacinth biomass; **(g–i)** [Edmim][Br]-pretreated water hyacinth biomass; **(j-l)** [Bmim][Br]-pretreated water hyacinth biomass; **(m–o)** [Hmim][Br]-pretreated water hyacinth biomass; **(p-r)** [Omim][Br]-pretreated water hyacinth biomass.

The water hyacinth (WH) biomass was subjected to different ILs pretreatment and samples were analyzed by SEM to observe the changes in the surface structures of raw cellulose and the pretreated celluloses, and recognizable difference between raw cellulose biomass and the treated cellulose biomass which are shown in Figures 4a–r with different levels of magnifications (500×, 1,000×, and 3,000×). Image analysis revealed highly fibrillar and intact morphology (Figures 4a–c) in case of untreated biomass, while the pretreated samples showed change in surface morphology and marked damage caused by the ILs (Figures 4d–r). Among all the pretreatments used, the [Emim][Br] was the highly effective pretreatment which have altered the structure of biomass to swollen and loose and fibrous structure has transformed into porous and amorphous form and been completely distorted (Figures 4d–f). Results shown in Figure 4 clearly supported the findings observed in case of FTIR and PXRD analysis. IL pretreatment using [Edmim][Br] had similar effects on water hyacinth biomass (Figures 4g–i) leading to maximum alterations in water hyacinth biomass structure after [Emim][Br] pretreatment. SEM analysis showed that following ILs [Omim][Br], [Hmim][Br] and [Bmim][Br] showed lesser effect on physical structure which was incapable of making any significant alterations. Microscopic shape of cellulose was smooth and compact, but the shapes of pretreated cellulose biomass were absolutely lamellar. So it is clear that due to smaller size of [Emim] cation, it interacts with the biomass more in comparison to the other. This may be due to the π-π stacking interaction of aromatic ring and hydrogen bonding at C-2 position. So–CH_3_ group are incorporated in [Edmim][Br], crystallinity and other degradation parameters are less. Moreover, when we are increasing the alkyl chain length etc. For example, [Bmim][Br], [Hmim][Br] and so on degradation of biomass is less. This might be due to the steric hindrance due to which imidazolium cannot interact with the biomass.

Structurally, cellulose is composed of the several cellulose strands run parallel and the large quantity of hydroxyls closed in a crystal cell, these parameters determine the CrI of raw cellulose. But after the treatment, change in microscopic structure of cellulose takes place that changes the CrI value. As a result, the pretreated cellulose biomass with ionic liquids had a looser microscopic shape. Our results are supported by Mood et al. (2014) where different sets of ionic liquids [Bmim][OTF], [MMIM][DMP], [BMIM][CL], [EMIM][DEP] and [EMIM][AC] are used to understand the physical and structural changes using SEM in barley straw.

### DSC analysis

The major components of biomass (cellulose, hemicellulose and lignin) degrade at different temperatures. Cellulose is highly crystalline, which makes it thermally stable. Hemicellulose and lignin on the other hand are amorphous and start to degrade before cellulose (Hill, 2006). Hemicelluloses are the least thermally stable components of biomass, due to the presence of acetyl groups (Bourgois et al., 1989). Lignin degrades partly over a wide temperature range, starting at relatively low temperatures (Nassar and MacKay, 2007).

DSC analysis was performed to determine the melt processing, which is due to change in the structural organization that influence the thermal behavior of cellulose, especially for its specific congregated structure. The DSC analysis validated the changes observed in the ILs pretreated biomass, including hydrogen bond strength, the CrI and microscopic changes. The thermal behavior can be assessed by TGA whilst the endothermic and exothermic reactions can be followed by DSC. The untreated and pretreated cellulose biomass was investigated by using DSC in a N_2_ atmosphere.

**Figure 5:**
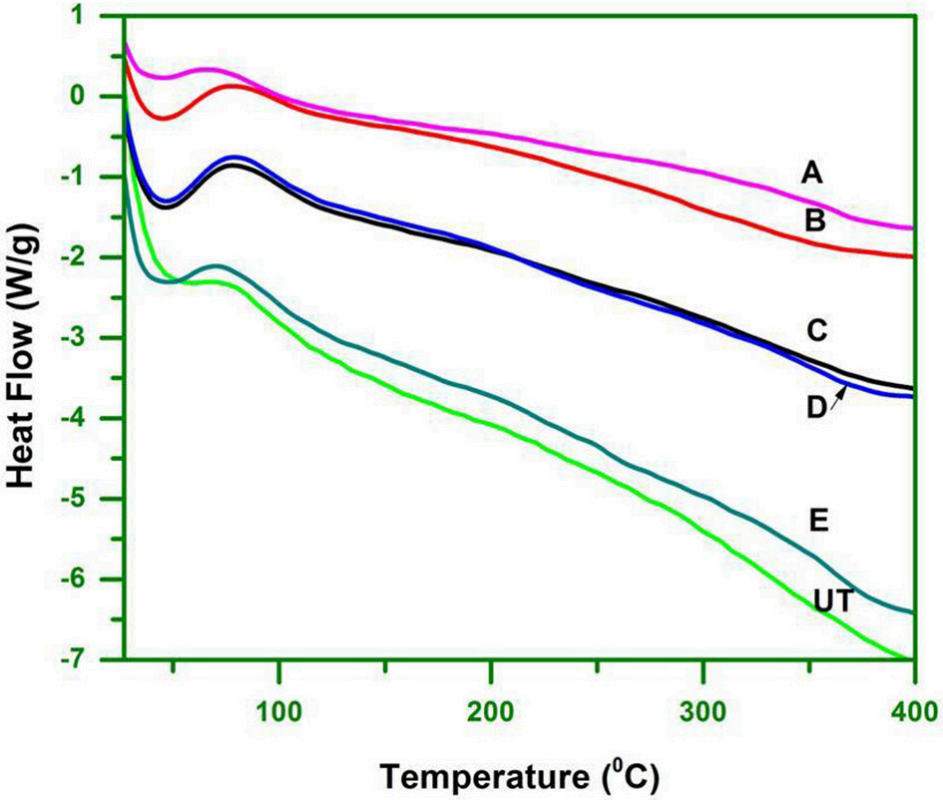
DSC curve that indicate the energy consumption property of cellulose of hyacinth biomass.

DSC curves showed only one clear endothermic change within the temperature range of 30–400°C (Figure 5). The endotherm that occurred from 30 to 100°C represented the loss of water to evaporation. Untreated WH had the highest evaporization temperature (T_eva_) of 54°C, followed by [Bmim][Br] treated WH at 44°C. The obtained results were due to the hydrophilic substances, such as hemicelluloses, lignin, and non-cellulosic materials that retain moisture in untreated WH. The DSC curves of treated WH were found to be 42°C, 45°C, 47°C, and 40°C.

### Mechanism of dissolution of lignin using ILs

Several studies reported that imidazolium based ILs could effectively dissolve complex lignocellulosic biomass network (Zhang et al., 2005; Erdmenger et al., 2008). The mechanism for lignin dissolution in ILs seems unlike that for cellulose dissolution. Xue et al. (2016), reported that the change of the solvent property for GVL-based binary solvent systems [Binary solvent systems consisting of biomass derived γ-valerolactone (GVL) and one co-solvent (e.g., water, ionic liquids, DMSO, and DMF)] was beneficial for the break of the strong hydrogen bonding in lignin and the combination of the aromatic nucleus and aliphatic chain regions, and thus resulted in much higher lignin solubility. Alvira et al. (2010) reported that ILs with anion activity (e.g., the 1-butyl-3 methyl imidazolium cation [C4mim]^+^) can dissolve the lignin and carbohydrate because ILs form hydrogen bonds between the non-hydrated chloride ions of the IL and the sugar hydroxyl protons in a 1:1 stoichiometry. As a result, the intricate network of non-covalent interactions among biomass polymers of cellulose, hemicellulose, and lignin is effectively disrupted while minimizing formation of degradation products (Alvira et al., 2010). Dissolution of lignin in ionic liquids is tremendously studied in which the effect of anions and their counter ion on the dissolution of lignin was reported by different workers. It is well-known that IL is fundamentally consisted of cation and anion parts. It is previously reported that nature of anions play a significant role in dissolving the lignin through hydrogen-bonding. While, imidazolium based cation has aromatic ring system which interacts with aromatic structural moieties (for instance paracoumaryl alcohol, coniferyl alcohol and synapyl alcohol) of lignin via π-π interaction (Hossain and Aldous, 2012). The effect of alkyl chain length of ILs on the lignin dissolution is not reported to the best of our knowledge. Hereby different imidazolium based ILs with tunable alkyl chain length (such as ethyl in [Emim]^+^and [Edmim]^+^, butyl in [Bmim]^+^, hexyl [Hmim]^+^ and octyl [Omim]^+^) but with the same counter anion Br^−^ are used in the present study. Therefore, the role of anion on the lignin dissolution can be neglected here. In addition, to understand the effect of acidic proton on the dissolution of lignin, C-2 position of imidazolium cation is substituted by methyl group in [Edmim][Br]. Mechanism of interaction between IL and sample can be described using the FESEM images and PXRD patterns of pretreated samples of WH, and it is found that [Emim][Br] IL is showing high crystallinity value while very less value of crystallinity index is in case of [Omim][Br]. The value of crystallinity index is gradually decreasing with alkyl chain length of ILs. In addition, similar trend was also obtained in FESEM images of the pretreatment samples. In FESEM images, sample pretreated with [Emim][Br] was found very much ruptured and inner layers were highly exposed and as a result fibril like structures were appeared (as shown in Figures 4m–o). Contrary to this, when the sample was pretreated with [Hmim] [Br] and [Omim] [Br] (Figures 4j–l,g–i), we did not find any noticeable changes than the untreated sample and fibril like structure was also absent. There are two ways through which IL is interacting with sample: a) via hydrogen bonding between acidic proton and lignin moiety and b) π-π interaction between imidazolium cation and aromatic groups of lignin that depends on the alkyl chain length at C-1 position of imidazolium cation (steric hindrance effect). Imidazolium based ionic liquids have significantly been used for synthesis of nano-materials specially in tuning the crystal phase and morphology of the nanoparticles. It has already been illustrated that during synthesis of nanoparticles, imidazolium based ionic liquids are bound at nucleation stage via aromatic π- (pi)-system or acidic proton via hydrogen bonding (Wang et al., 2008). Similarly, biomolecules often consist of aromatic system containing molecules like protein, amino acids, polypeptide chains, lignin, hemicellulose and so on. Therefore, the possibility of interaction between biomolecules and ionic liquid is increased. As a result, effect of ionic liquid on the biological system can also be noticed. Thus, from detailed studies of all pretreated samples, it was noticed that the alkyl chain length of ILs is playing a significant role in interaction which is to be occurred between ILs and lignin. It means smaller the pendant alkyl chain length greater would be the binding of the ILs on the surface of pretreated sample. As the alkyl chain length increases, binding of ILs on the surface of samples decreases resulting of lignin dissolution was found very less as in case of [Omim][Br] IL. In order to see the effect of acidic proton on C-2 position of IL, [Edmim][Br] IL was employed for pretreatment of WH biomass sample; in that case crystallinity index was found to be increased to noticeable extent because crystallinity is strongly influenced by the lignocellulosic biomass complex composition. The raw lignocellulosic biomass has a lowest crystallinity because it has a higher content of lignin and hemicellulose which are amorphous in nature (Xu et al., 2007). In this study, the Crl of the untreated WH biomass was higher than that of the untreated WH biomass. This indicates that the higher value of crystallinity results from modification of the complex composition of the pretreated WH biomass. In other word, substitution of acidic proton by methyl group is also decreasing the attachment of the [Edmim][Br] on the surface of pretreated WH biomass sample due to steric hindrance effect (shown in Table 4). Perez-Pimienta et al. (2016) confirmed that [Emim][OAc] readily dissolved lignin in the tested biomass. The authors then compared the solubility of lignin in ILs with the same anion [OAc], but with different cations [Emim]^+^, [Bmim]^+^, [N_4448_]^+^, [Bm_2_im]^+^, and [Bpyr]^+^, and noted that the solubility follows [Emim]^+^ = [Bmim]^+^ = [Bpyr]^+^ > [Bm_2_im]^+^ >[N_4448_]^+^.

## Conclusions

In summary, we have succeeded to develop an IL based methodology for lignin solubilization from WH, whose biomass is one of the most important feedstock for the production of fermentable sugar and bio-ethanol. Amongst 5 different imidazolium based ILs, 1-ethyl-3-methylimidazolium bromide [Emim][Br] is found most efficient in biomass conversion followed by 1-ethyl-2,3-dimethylimidazolium bromide [Edmim][Br]. Analysis reveals that imidazolium cation with lower alkyl chain length like [Emim]^+^can interact with the aromatic rings of lignin moieties via π stacking as well as H-bonding. However, ILs with higher alkyl chain length cannot interact so efficiently with the lignin due to steric hindrance and causes less dissolution. To the best of our knowledge, the effect of IL cation on the hydrolysis of WH biomass is not reported so far and can pave the way for an IL based promising approach for hydrolysis of other biomasses.

## Author contributions

JS and RS have contributed equally to this manuscript under the guidance of AK and PG. PG and AK prepared and edited the manuscript. MK suggested some improvements to the manuscript.

### Conflict of interest statement

The authors declare that the research was conducted in the absence of any commercial or financial relationships that could be construed as a potential conflict of interest.

## Abbreviations

FTIR: Fourier transform infrared
XRD: X-ray diffraction
SEM: scanning electron microscope
DSC: differential scanning calorimeter
CrI: crystallinity index
